# The genome sequence of an ichneumonid wasp, *Oxytorus armatus *Thomson, 1883

**DOI:** 10.12688/wellcomeopenres.21215.1

**Published:** 2024-04-12

**Authors:** Gavin R. Broad, Chris Fletcher, Inez Januszczak, Natural History Museum Genome Acquisition Lab, Darwin Tree of Life Barcoding collective, Wellcome Sanger Institute Tree of Life Management, Samples and Laboratory team, Wellcome Sanger Institute Scientific Operations: Sequencing Operations, Wellcome Sanger Institute Tree of Life Core Informatics team, Tree of Life Core Informatics collective, Darwin Tree of Life Consortium

**Affiliations:** 1 Natural History Museum, London, England, UK

**Keywords:** Oxytorus armatus, ichneumonid wasp, genome sequence, chromosomal, Hymenoptera

## Abstract

We present a genome assembly from an individual male *Oxytorus armatus* (an ichneumonid wasp; Arthropoda; Insecta; Hymenoptera; Ichneumonidae). The genome sequence is 367.8 megabases in span. Most of the assembly is scaffolded into 13 chromosomal pseudomolecules. The mitochondrial genome has also been assembled and is 56.22 kilobases in length.

## Species taxonomy

Eukaryota; Opisthokonta; Metazoa; Eumetazoa; Bilateria; Protostomia; Ecdysozoa; Panarthropoda; Arthropoda; Mandibulata; Pancrustacea; Hexapoda; Insecta; Dicondylia; Pterygota; Neoptera; Endopterygota; Hymenoptera; Apocrita; Ichneumonoidea; Ichneumonidae; Oxytorinae; *Oxytorus*; *Oxytorus armatus* Thomson, 1883 (NCBI:txid495374).

## Background

*Oxytorus armatus* is a widespread ichneumonid wasp. Females are rather distinctive as they have white-striped antennae, small projections on the propodeum (apophyses), a long and posteriorly compressed metasoma (the abdomen beyond the first segment), and short, wide ovipositor sheaths. The only other European (including British) species of *Oxytorus*, *O. luridator*, can be separated most easily by its much smaller apophyses, closed areolet in the fore wing, and shorter metasoma. Males are very similar but can usually be separated by the absence of the areolet in *O. armatus* and the rougher, more ‘leathery’ sculpture on the second metasomal tergite. Kerrich (1939) and van Rossem (1987) provide some notes on identification. The main problem in identifying males of *Oxytorus* is in recognising that they are oxytorines. The subfamily Oxytorinae comprises the single genus *Oxytorus*, which is defined by some distinctive features such as the female metasoma shape, the flattened clypeus and the exceptionally long maxillary palps. Males, however, look rather indistinct and are often passed over as specimens of Ctenopelmatinae, Cryptinae or Phygadeuontinae.

Based on specimens in museum collections, especially light-trapped individuals identified by GRB, adults are active from June to early September, most commonly in June and July. *Oxytorus armatus* has a wide range across Europe, as does *O. luridator*, although *O. luridator* is perhaps the more commonly collected of the two. Usually found in deciduous woodland, we know very little about the ecology of *O. armatus*. As with *O. luridator*, males are frequently attracted to light so are presumably at least partly nocturnal or crepuscular. In fact, the small subfamily Oxytorinae is one of only three ichneumonid subfamilies entirely lacking host records. Oxytorinae is the most species-rich of those three, with 25 described species and several undescribed species found across both temperate and tropical areas of Asia, Europe and the Americas ( Broad *et al.*, 2018; Riedel *et al.*, 2021). As pointed out by Wahl (1990), when classifying *Oxytorus* in their own subfamily, the short ovipositor of *Oxytorus* has a subapical notch, which means they will be endoparasitoids of larvae.

In the few large-scale phylogenetic studies which have included *Oxytorus* ( Bennett *et al.*, 2019; Quicke *et al.*, 2009; Sharanowski *et al.*, 2021), they have been found to be part of a grouping called the ‘ophioniformes’, and part of a very large clade which are invariably koinobiont endoparasitoids of holometabolous insect larvae, i.e., the host develops further following oviposition, with the wasp larva feeding internally. *Oxytorus* might be closely related to some of the groups of sawfly parasitoids currently classified as Ctenopelmatinae, which are not monophyletic *Oxytorus* ( Bennett *et al.*, 2019; Quicke *et al.*, 2009; Sharanowski *et al.*, 2021), but are all parasitoids of sawfly (Hymenoptera) larvae. It is conceivable that *Oxytorus* will be found to be parasitoids of sawfly larvae, but it is striking that while the larvae of many northern European sawfly species have been reared, *Oxytorus* never has. The metasoma adaptations, and the fact that females seem to stay near ground level ( Broad *et al.*, 2018) suggests that the hosts will be concealed in loose, or easily penetrated substrate. The availability of a genome will help to pin down the affinities of this enigmatic group of Darwin Wasps, and hopefully spur efforts to rear these wasps.

## Genome sequence report

**Figure 1.**
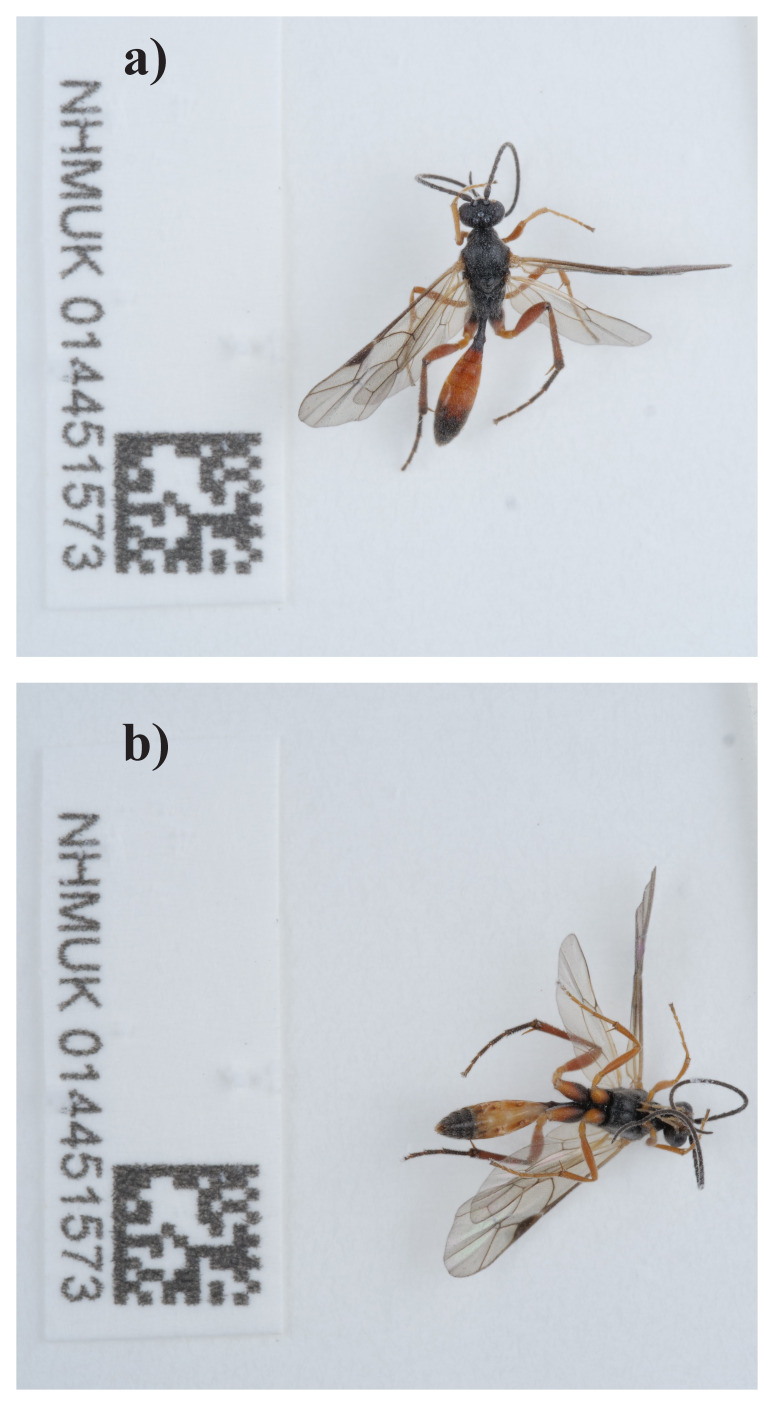
Photographs of the *Oxytorus armatus* (iyOxyArmt1) specimen used for genome sequencing.

The genome was sequenced from one male *Oxytorus armatus* ( Figure 1) collected from Bert’s Pheasant Pen, Wytham Woods, Oxfordshire, UK (51.77, –1.31). A total of 62-fold coverage in Pacific Biosciences single-molecule HiFi long reads was generated. Primary assembly contigs were scaffolded with chromosome conformation Hi-C data. Manual assembly curation corrected 35 missing joins or mis-joins, reducing the scaffold number by 26.42%, and increasing the scaffold N50 by 0.66%.

**Figure 2.**
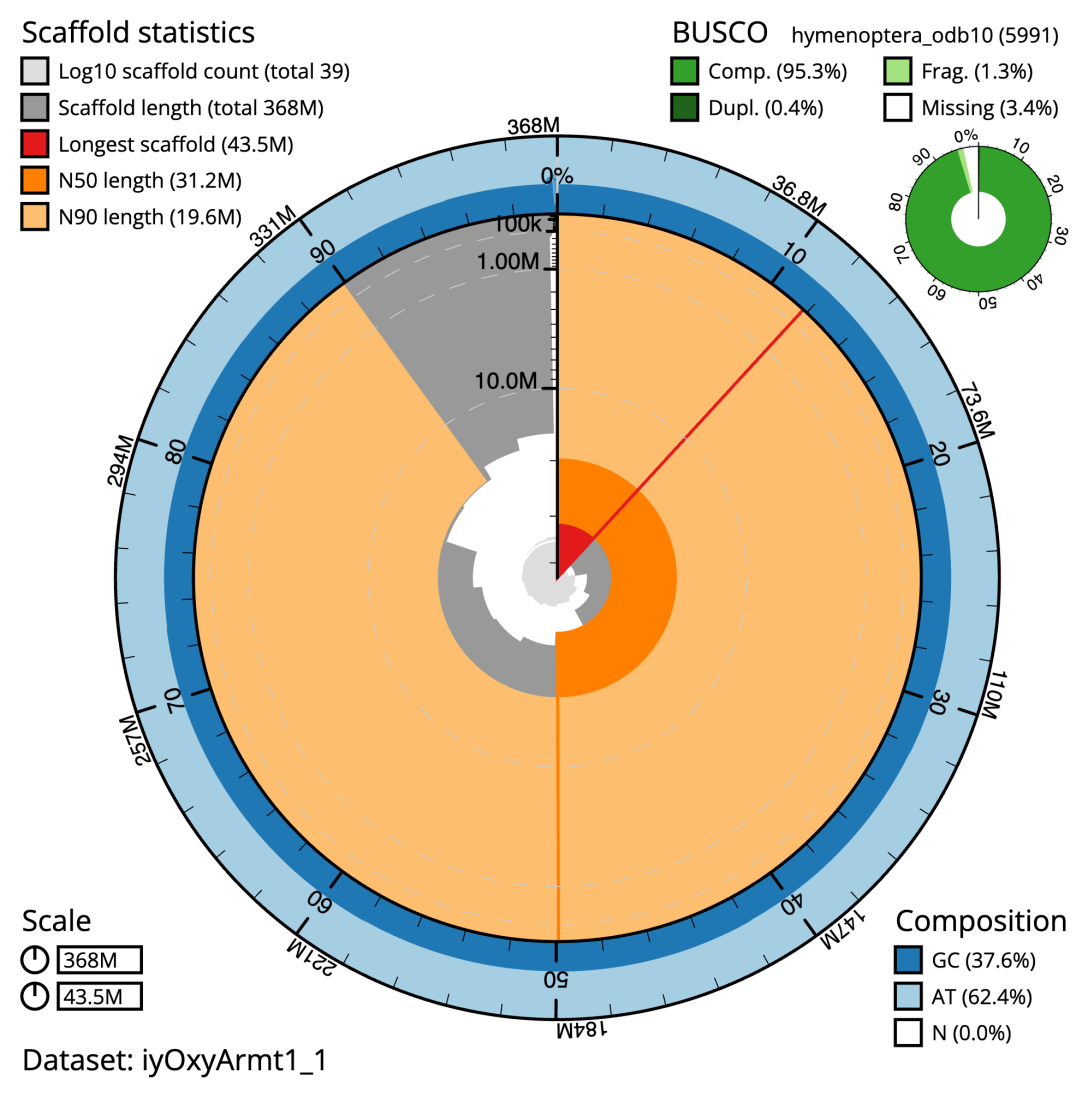
Genome assembly of *Oxytorus armatus*, iyOxyArmt1.1: metrics. The BlobToolKit snail plot shows N50 metrics and BUSCO gene completeness. The main plot is divided into 1,000 size-ordered bins around the circumference with each bin representing 0.1% of the 367,853,913 bp assembly. The distribution of scaffold lengths is shown in dark grey with the plot radius scaled to the longest scaffold present in the assembly (43,478,420 bp, shown in red). Orange and pale-orange arcs show the N50 and N90 scaffold lengths (31,210,731 and 19,609,202 bp), respectively. The pale grey spiral shows the cumulative scaffold count on a log scale with white scale lines showing successive orders of magnitude. The blue and pale-blue area around the outside of the plot shows the distribution of GC, AT and N percentages in the same bins as the inner plot. A summary of complete, fragmented, duplicated and missing BUSCO genes in the hymenoptera_odb10 set is shown in the top right. An interactive version of this figure is available at https://blobtoolkit.genomehubs.org/view/iyOxyArmt1_1/dataset/iyOxyArmt1_1/snail.

**Figure 3.**
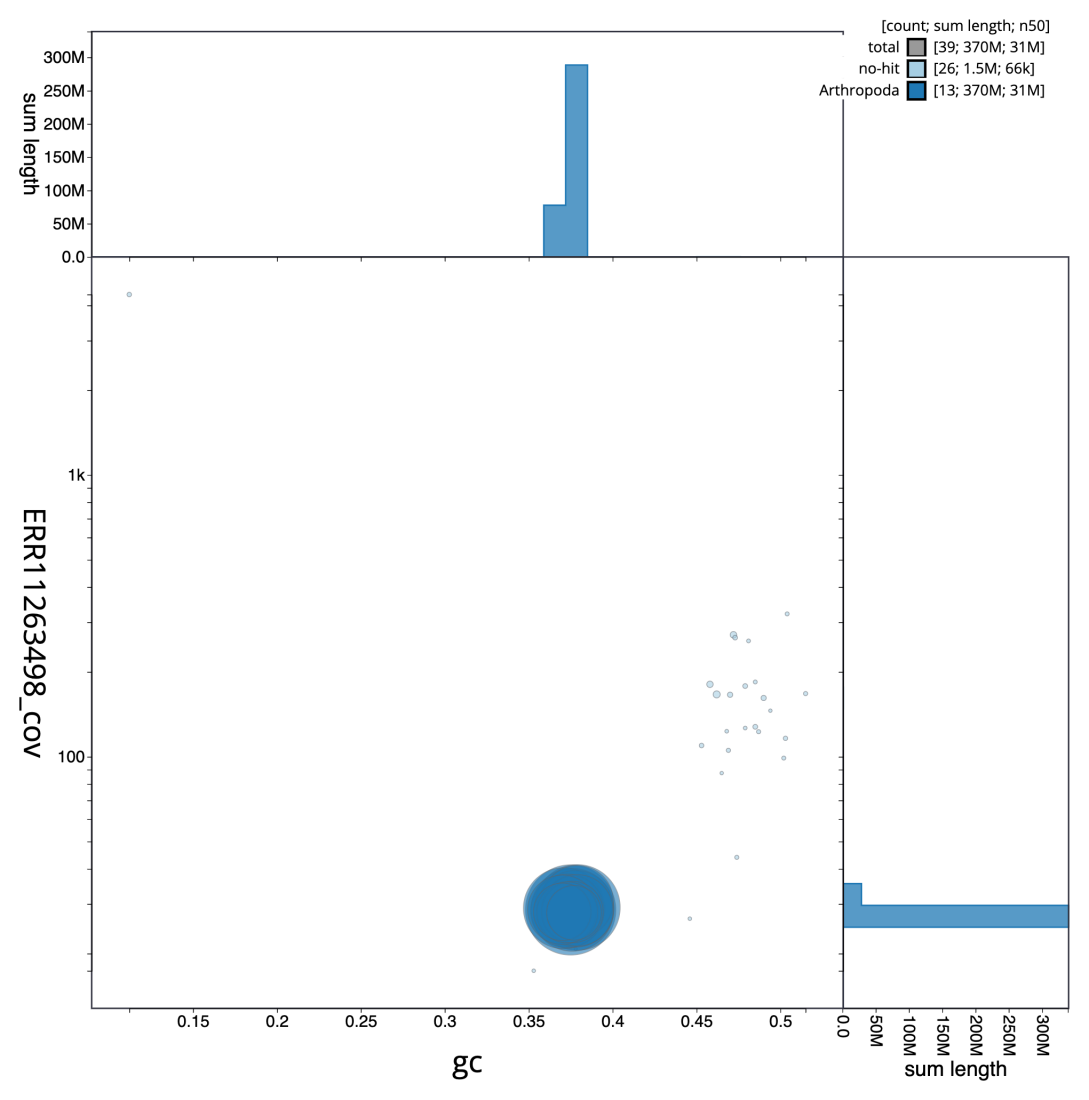
Genome assembly of *Oxytorus armatus*, iyOxyArmt1.1: BlobToolKit GC-coverage plot. Sequences are coloured by phylum. Circles are sized in proportion to sequence length. Histograms show the distribution of sequence length sum along each axis. An interactive version of this figure is available at https://blobtoolkit.genomehubs.org/view/iyOxyArmt1_1/dataset/iyOxyArmt1_1/blob.

**Figure 4.**
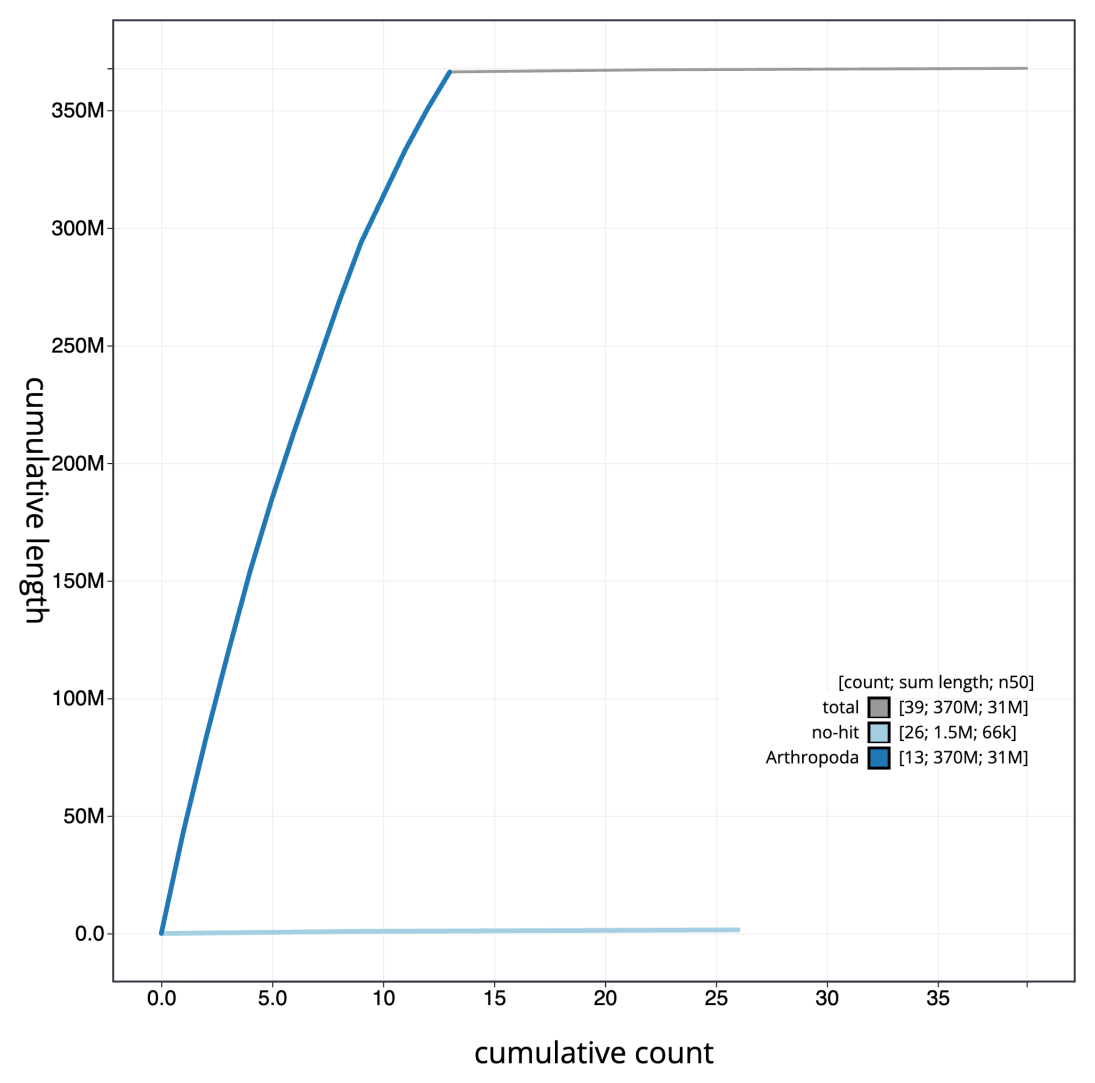
Genome assembly of *Oxytorus armatus*, iyOxyArmt1.1: BlobToolKit cumulative sequence plot. The grey line shows cumulative length for all sequences. Coloured lines show cumulative lengths of sequences assigned to each phylum using the buscogenes taxrule. An interactive version of this figure is available at https://blobtoolkit.genomehubs.org/view/iyOxyArmt1_1/dataset/iyOxyArmt1_1/cumulative.

**Figure 5.**
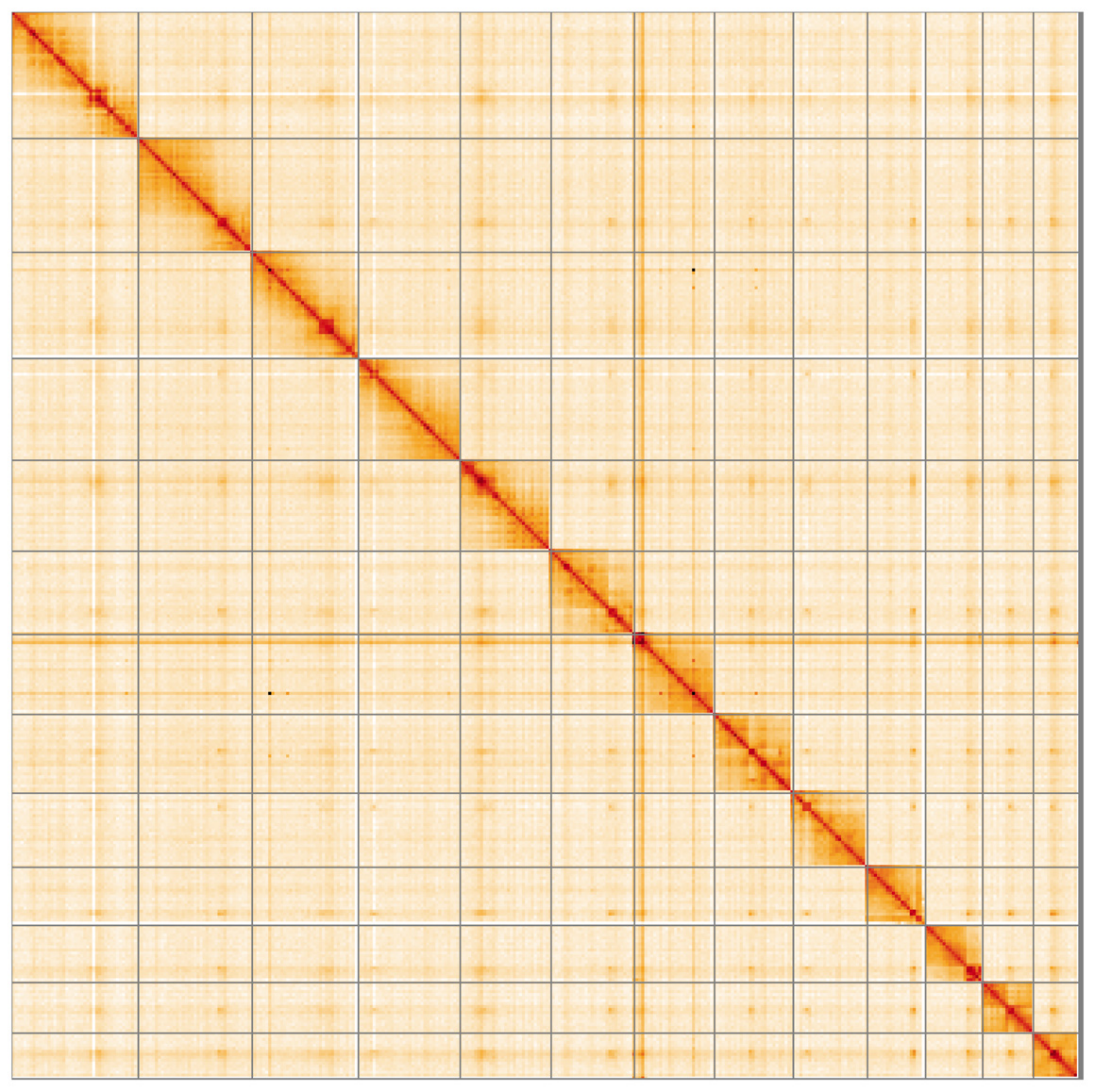
Genome assembly of *Oxytorus armatus*, iyOxyArmt1.1: Hi-C contact map of the iyOxyArmt1.1 assembly, visualised using HiGlass. Chromosomes are shown in order of size from left to right and top to bottom. An interactive version of this figure may be viewed at https://genome-note-higlass.tol.sanger.ac.uk/l/?d=QG7ssJ0OSkGxmzq4ZBVN8A.

The final assembly has a total length of 367.8 Mb in 38 sequence scaffolds with a scaffold N50 of 31.2 Mb ( Table 1). The snail plot in Figure 2 provides a summary of the assembly statistics, while the distribution of assembly scaffolds on GC proportion and coverage is shown in Figure 3. The cumulative assembly plot in Figure 4 shows curves for subsets of scaffolds assigned to different phyla. Most (99.98%) of the assembly sequence was assigned to 13 chromosomal-level scaffolds. The genome is of a haploid male specimen. Chromosome-scale scaffolds confirmed by the Hi-C data are named in order of size ( Figure 5; Table 2). The mitochondrial genome was also assembled and can be found as a contig within the multifasta file of the genome submission.

**Table 1.** Genome data for *Oxytorus armatus*, iyOxyArmt1.1.

Project accession data		
Assembly identifier	iyOxyArmt1.1	
Species	*Oxytorus armatus*	
Specimen	iyOxyArmt1	
NCBI taxonomy ID	495374	
BioProject	PRJEB61495	
BioSample ID	SAMEA14448319	
Isolate information	iyOxyArmt1, male (DNA and Hi-C sequencing)	
Assembly metrics *		*Benchmark*
Consensus quality (QV)	67.8	*≥ 50*
*k*-mer completeness	100.0%	*≥ 95%*
BUSCO **	C:95.3%[S:94.9%,D:0.4%], F:1.3%,M:3.4%,n:5,991	*C ≥ 95%*
Percentage of assembly mapped to chromosomes	99.98%	*≥ 95%*
Sex chromosomes	None	*localised homologous pairs*
Organelles	Mitochondrial genome: 56.22 kb	*complete single alleles*
Raw data accessions		
PacificBiosciences SEQUEL II	ERR11263498	
Hi-C Illumina	ERR11271517	
Genome assembly		
Assembly accession	GCA_958009045.1	
Span (Mb)	367.8	
Number of contigs	357	
Contig N50 length (Mb)	2.0	
Number of scaffolds	38	
Scaffold N50 length (Mb)	31.2	
Longest scaffold (Mb)	43.48	

* Assembly metric benchmarks are adapted from column VGP-2020 of “Table 1: Proposed standards and metrics for defining genome assembly quality” from Rhie *et al.* (2021). ** BUSCO scores based on the hymenoptera_odb10 BUSCO set using version 5.3.2. C = complete [S = single copy, D = duplicated], F = fragmented, M = missing, n = number of orthologues in comparison. A full set of BUSCO scores is available at https://blobtoolkit.genomehubs.org/view/iyOxyArmt1_1/dataset/iyOxyArmt1_1/busco.

**Table 2.** Chromosomal pseudomolecules in the genome assembly of *Oxytorus armatus*, iyOxyArmt1.

INSDC accession	Chromosome	Length (Mb)	GC%
OY253744.1	1	43.48	37.5
OY253745.1	2	39.08	38.0
OY253746.1	3	36.46	37.5
OY253747.1	4	34.94	37.0
OY253748.1	5	31.21	38.0
OY253749.1	6	27.47	38.0
OY253750.1	7	28.59	37.5
OY253751.1	8	27.05	38.0
OY253752.1	9	25.44	37.0
OY253753.1	10	19.94	37.5
OY253754.1	11	19.61	37.5
OY253755.1	12	17.39	37.0
OY253756.1	13	15.67	37.5
OY253757.1	MT	0.06	11.5

The estimated Quality Value (QV) of the final assembly is 67.8 with *k*-mer completeness of 100.0%, and the assembly has a BUSCO v5.3.2 completeness of 95.3% (single = 94.9%, duplicated = 0.4%), using the hymenoptera_odb10 reference set ( *n* = 5,991).

Metadata for specimens, barcode results, spectra estimates, sequencing runs, contaminants and pre-curation assembly statistics are given at https://links.tol.sanger.ac.uk/species/495374.

## Methods

### Sample acquisition and nucleic acid extraction

A male *Oxytorus armatus* (specimen ID NHMUK014451573, ToLID iyOxyArmt1) was collected from Wytham Woods, Bert's Pheasant Pen, Wytham Woods, Oxfordshire (biological vice-county Berkshire), UK (latitude 51.77, longitude –1.31) on 2021-09-02, using an aerial net. The specimen was collected by Gavin Broad, Chris Fletcher and Inez Januszczak (Natural History Museum) and identified by Gavin Broad, and then preserved by dry freezing at –80 °C.

The workflow for high molecular weight (HMW) DNA extraction at the Wellcome Sanger Institute (WSI) includes a sequence of core procedures: sample preparation; sample homogenisation, DNA extraction, fragmentation, and clean-up. The sample was prepared for DNA extraction at the WSI Tree of Life Core Laboratory: the iyOxyArmt1 sample was weighed and dissected on dry ice ( Jay *et al.*, 2023). Tissue from the whole organism was homogenised using a PowerMasher II tissue disruptor ( Denton *et al.*, 2023a).

HMW DNA was extracted in the WSI Scientific Operations core using the Automated MagAttract v2 protocol ( Oatley *et al.*, 2023). The DNA was sheared into an average fragment size of 12–20 kb in a Megaruptor 3 system with speed setting 31 ( Bates *et al.*, 2023). Sheared DNA was purified by solid-phase reversible immobilisation ( Strickland *et al.*, 2023): in brief, the method employs a 1.8X ratio of AMPure PB beads to sample to eliminate shorter fragments and concentrate the DNA. The concentration of the sheared and purified DNA was assessed using a Nanodrop spectrophotometer and Qubit Fluorometer and Qubit dsDNA High Sensitivity Assay kit. Fragment size distribution was evaluated by running the sample on the FemtoPulse system.

Protocols developed by the WSI Tree of Life laboratory are publicly available on protocols.io ( Denton *et al.*, 2023b).

### Sequencing

Pacific Biosciences HiFi circular consensus DNA sequencing libraries were constructed according to the manufacturers’ instructions. DNA sequencing was performed by the Scientific Operations core at the WSI on a Pacific Biosciences SEQUEL II instrument. Hi-C data were also generated from remaining tissue of iyOxyArmt1 using the Arima2 kit and sequenced on the Illumina NovaSeq 6000 instrument.

### Genome assembly, curation and evaluation

Assembly was carried out with Hifiasm ( Cheng *et al.*, 2021). The assembly was then scaffolded with Hi-C data ( Rao *et al.*, 2014) using YaHS ( Zhou *et al.*, 2023). The assembly was checked for contamination and corrected as described previously ( Howe *et al.*, 2021). Manual curation was performed using HiGlass ( Kerpedjiev *et al.*, 2018) and PretextView ( Harry, 2022). The mitochondrial genome was assembled using MitoHiFi ( Uliano-Silva *et al.*, 2023) and OATK ( Zhou, 2023).

A Hi-C map for the final assembly was produced using bwa-mem2 ( Vasimuddin *et al.*, 2019) in the Cooler file format ( Abdennur & Mirny, 2020). To assess the assembly metrics, the *k*-mer completeness and QV consensus quality values were calculated in Merqury ( Rhie *et al.*, 2020). This work was done using Nextflow ( Di Tommaso *et al.*, 2017) DSL2 pipelines “sanger-tol/readmapping” ( Surana *et al.*, 2023a) and “sanger-tol/genomenote” ( Surana *et al.*, 2023b). The genome was analysed within the BlobToolKit environment ( Challis *et al.*, 2020) and BUSCO scores ( Manni *et al.*, 2021; Simão *et al.*, 2015) were calculated.

Table 3 contains a list of relevant software tool versions and sources.

**Table 3.** Software tools: versions and sources.

Software tool	Version	Source
BlobToolKit	4.2.1	https://github.com/blobtoolkit/blobtoolkit
BUSCO	5.3.2	https://gitlab.com/ezlab/busco
Hifiasm	0.16.1	https://github.com/chhylp123/hifiasm
HiGlass	1.11.6	https://github.com/higlass/higlass
Merqury	MerquryFK	https://github.com/thegenemyers/MERQURY.FK
MitoHiFi	3.01	https://github.com/marcelauliano/MitoHiFi
OATK	-	https://github.com/c-zhou/oatk
PretextView	0.2	https://github.com/wtsi-hpag/PretextView
sanger-tol/genomenote	v1.0	https://github.com/sanger-tol/genomenote
sanger-tol/readmapping	1.1.0	https://github.com/sanger-tol/readmapping/tree/1.1.0
YaHS	1.1a.2	https://github.com/c-zhou/yahs

### Wellcome Sanger Institute – Legal and Governance

The materials that have contributed to this genome note have been supplied by a Darwin Tree of Life Partner. The submission of materials by a Darwin Tree of Life Partner is subject to the **‘Darwin Tree of Life Project Sampling Code of Practice’**, which can be found in full on the Darwin Tree of Life website here. By agreeing with and signing up to the Sampling Code of Practice, the Darwin Tree of Life Partner agrees they will meet the legal and ethical requirements and standards set out within this document in respect of all samples acquired for, and supplied to, the Darwin Tree of Life Project.

Further, the Wellcome Sanger Institute employs a process whereby due diligence is carried out proportionate to the nature of the materials themselves, and the circumstances under which they have been/are to be collected and provided for use. The purpose of this is to address and mitigate any potential legal and/or ethical implications of receipt and use of the materials as part of the research project, and to ensure that in doing so we align with best practice wherever possible. The overarching areas of consideration are:

•     Ethical review of provenance and sourcing of the material

•     Legality of collection, transfer and use (national and international)

Each transfer of samples is further undertaken according to a Research Collaboration Agreement or Material Transfer Agreement entered into by the Darwin Tree of Life Partner, Genome Research Limited (operating as the Wellcome Sanger Institute), and in some circumstances other Darwin Tree of Life collaborators.

## Data Availability

European Nucleotide Archive: *Oxytorus armatus*. Accession number PRJEB61495; https://identifiers.org/ena.embl/PRJEB61495 ( Wellcome Sanger Institute, 2023). The genome sequence is released openly for reuse. The *Oxytorus armatus* genome sequencing initiative is part of the Darwin Tree of Life (DToL) project. All raw sequence data and the assembly have been deposited in INSDC databases. The genome will be annotated using available RNA-Seq data and presented through the Ensembl pipeline at the European Bioinformatics Institute. Raw data and assembly accession identifiers are reported in Table 1.

## Author information

Members of the Natural History Museum Genome Acquisition Lab are listed here: https://doi.org/10.5281/zenodo.7139035.

Members of the Darwin Tree of Life Barcoding collective are listed here: https://doi.org/10.5281/zenodo.4893703.

Members of the Wellcome Sanger Institute Tree of Life Management, Samples and Laboratory team are listed here: https://doi.org/10.5281/zenodo.10066175.

Members of Wellcome Sanger Institute Scientific Operations: Sequencing Operations are listed here: https://doi.org/10.5281/zenodo.10043364.

Members of the Wellcome Sanger Institute Tree of Life Core Informatics team are listed here: https://doi.org/10.5281/zenodo.10066637.

Members of the Tree of Life Core Informatics collective are listed here: https://doi.org/10.5281/zenodo.5013541.

Members of the Darwin Tree of Life Consortium are listed here: https://doi.org/10.5281/zenodo.4783558.
