# Supplementary material for: The genome sequence of an ichneumonid wasp, Oxytorus armatus Thomson, 1883
Source: Wellcome Open Res. 2024 Apr 12;9:190. [Version 1] doi: 10.12688/wellcomeopenres.21215.1 (PMC11380729; doi:10.12688/wellcomeopenres.21215.1)
Supplement: Peer review history

# Reviewer report: Reviewer response for version 1

**DOI:** 10.21956/wellcomeopenres.23462.r94872

**Published:** 2024-09-07

**Authors:** Cheng Sun

**Affiliations:** 1 Capital Normal University, Beijing, Beijing, China

The study presented a chromosome-level genome assembly for a widespread ichneumonid wasp, *Oxytorus armatus, *using genomic DNAs obtained from one single male. The genome sequence is 367.8 Mb in length, with 13 chromosomal pseudomolecules. The Method of this paper was clear and the Result was reliable. This reference genome will be useful in understanding the evolution and rearing of this wasp.

Are sufficient details of methods and materials provided to allow replication by others?

Yes

Is the rationale for creating the dataset(s) clearly described?

Yes

Are the datasets clearly presented in a useable and accessible format?

Yes

Are the protocols appropriate and is the work technically sound?

Yes

Reviewer Expertise:

insect genome evolution

I confirm that I have read this submission and believe that I have an appropriate level of expertise to confirm that it is of an acceptable scientific standard.

# Reviewer report: Reviewer response for version 1

**DOI:** 10.21956/wellcomeopenres.23462.r94871

**Published:** 2024-09-01

**Authors:** Juan Wulff

**Affiliations:** 1 Department of Entomology and Plant Pathology, North Carolina State University, Raleigh, USA

The work in general is very good and the method used for sequencing is quite robust. The manuscript is short, but I understand that it is more of a report or short communication than a research paper. This is my fourth time reviewing this type of work for this journal and I always mention the same thing, especially since they are all almost identical, the main weakness of these studies is that the annotation was done using a database without transcriptomes to support gene annotation. However, I must say that given the large scale of this project, obtaining the genomes of closely related species will improve gene annotation. Likewise, the absence of species-specific isoforms will always be a weakness of this study and other similar ones that I have reviewed. Finally, I think that the quality of the genome assembly is very good.

Are sufficient details of methods and materials provided to allow replication by others?

Yes

Is the rationale for creating the dataset(s) clearly described?

Yes

Are the datasets clearly presented in a useable and accessible format?

Yes

Are the protocols appropriate and is the work technically sound?

Yes

Reviewer Expertise:

Biology, Entomology, Molecular biology and Genetics.

I confirm that I have read this submission and believe that I have an appropriate level of expertise to confirm that it is of an acceptable scientific standard.
